# Sustainable indigenous bio-mixture for restoration the soil point source pollution with special reference to chlorpyrifos

**DOI:** 10.1007/s10661-024-12494-5

**Published:** 2024-03-13

**Authors:** Ayman H. Mansee, Amal M. Ebrahim, Essam A. Koreish

**Affiliations:** 1 Department of Pesticide Chemistry & Technology, Faculty of Agriculture, Alexandria University, Alexandria, Egypt; 2 Department of Soil & Water Science, Faculty of Agriculture, Alexandria University, Alexandria, Egypt

**Keywords:** Land degradation neutrality, Chlorpyrifos, Biomixture, Vermicompost, NPK, Point source pollution

## Abstract

Improper pesticide handling is the main cause of contamination of the environment in agricultural systems. This could be caused by leakage of spraying liquid, leftovers, and inappropriate washing of spraying equipment. This study assessed the ability of suggested biomixture modules for remediate repetitive cycles of high chlorpyrifos doses. In three consecutive treatments, four tested modules were contaminated with 160 µg g^−1^ chlorpyrifos. Chlorpyrifos residues, dehydrogenase activity, and microbial respiration were continuously monitored for 22 weeks. Six bacterial consortia were isolated at the end of the experiment from four treated modules (B^+3^, BF^+3^, S^+3^, and SF^+3^) and two from untreated modules (B and S). The isolated consortium efficiency in degrading chlorpyrifos was studied. The results revealed that the best chlorpyrifos removal efficiency was achieved when using the stimulated biomixture module (BF) recorded 98%, 100%, and 89%, at the end of three chlorpyrifos treatments, respectively. Such removal efficiency was compatible with the biological activity results of the tested modules: dehydrogenase activity and microbial respiration. There was no difference in the efficiency among the S, B, and BF^+3^ consortia. The results presented here demonstrate that the combination of vermicompost, wheat straw, soil, and NPK (stimulated biomixture module) can successfully reduce the risk of a point source of pesticide pollution.

## Introduction

In order to alleviate water stress and enhance sustainability, European countries have established a target for remediation and safe reusing wastewater by 2030. Thus, wastewater remediation should be a worldwide adapting target as well as minimizing pollution in pesticide application spots (Angelakis et al., 2018; Yadav et al., 2022). To ensure cleaner production in agriculture, the main concern is to use biotechnological innovations that are environmentally friendly (Jalali et al., 2023). Numerous physicochemical approaches have been researched for the treatment of pesticide polluted environmental phases (water, sediments, and soils); these techniques are costly and can contaminate the medium with additional harmful contaminants (John et al., 2015). Bioremediation according to Kour et al. (2021) and Yadav et al. (2022) is a solution that can result in the degradation or transformation of environmental pollutants into harmless or less hazardous forms. It is also non-invasive and less expensive than conventional approaches. Additionally, Raghunandan et al. (2018) proposed that bioremediation is a revolutionary technology that ought to be developed to shield people and the environment from the negative effects of environmental pollution. Furthermore, bioremediation has become a relatively inexpensive technique; bioremediation can be a useful tool in mitigating the impacts of pollution and making contaminated soil less polluted and toxic-free.

A series of studies were carried out by Mansee et al., (2000, 2017a, b) with the aim of monitoring the ability of (1) genetically engineered *Escherichia coli*; (2) five bacterial strains isolated from mud waste of a gas station in Alexandria, Egypt; and (3) four bacterial strains identified from two types of soil with varying histories of atrazine applications, as a bioremediation tool to remediate coumaphos, methyl-parathion or paraoxon, and atrazine, respectively. In another study, Abdelgawad et al. (2022) isolated three individual bacterial strains from two types of soils with a different history of atrazine applications; the molecular method identified them as *Stenotrophomonas* sp., *Bacillus cereus*, and *Paenarthrobacter ureafaciens*. Researchers (Abdelgawad et al., 2022) looked at how several factors, such as mixing, starving, UV exposure, and sodium citrate, could improve the atrazine bioremediation process. They observed, first, that the atrazine degradation percentage was 61.39% and 36.59% when using both *Paenarthrobacter ureafaciens* and *Bacillus cereus*, respectively. Second, following the starvation procedure, the atrazine degradation efficacy for *Stenotrophomonas* sp., *Bacillus cereus*, and *Paenarthrobacter ureafaciens* rose by 1.28- to 4.32-fold, and third, the studied isolates’ individual or mixture efficiencies increased from 1.08 to 4.63 times as a result of the UV exposure.

Coppola et al. (2011), Mukherjee et al. (2016), Góngora-Echeverría et al. (2018), Adak et al. (2020), and Fernandez et al. (2022) conducted several studies that suggest that multiple technologies should be combined in certain cases to achieve satisfactory results. These studies suggest that the biobed system is a biological technology designed to prevent pollution with pesticides by percolating it over a bioactive matrix (biomixture), which is able to sorbed and degraded the pollutants. They also emphasize that the composition of the biomixtures is dependent on the availability of agro-industrial wastes in the area, and that each county should have its suitable design.

Chin-Pampillo et al., (2015a, b) prepared five biomixtures by mixing different lignocellulosic materials with its compost or peat and soil to assess the dissipation rate of the carbofuran. They found that better detoxification capacity occurred when the compost-based biomixtures were used. Castillo-Diaz et al. (2016) state that adding vermicomposting to the biobed systems can lessen the negative environmental effects while promoting the sorption and degradation of the pesticides by stimulating microbial activity. The biomixture developed by Romero et al. (2019) which consisted of topsoil, vermicompost, and winery trash (w-biomixture) was used to investigate its potential for three tested pesticides’ dissipation and their effects on biomixture microbial activities, compared to the original biomixture (soil, peat, and straw). They found that the innovative w-biomixture has a high prospective pesticide removal capability as an alternate model to the original one. Fernandez et al. (2022) developed a biomixture of wheat and soil to assess its effectiveness in adsorbing and degrading 2,4-dichlorophenoxyacetic acid (2,4-D) and 2,4-dichlorophenol (2,4-DCP). After 15 days, they came to the conclusion that the biomixture had successfully broken down 2,4-D, with a removal efficacy of more than 96%, and complete dissipation of the 2,4-DCP. Lescano et al. (2022) constructed an experimental biobed to handle wastewater that was contaminated with carbendazim, imidacloprid, prometryn, atrazine, and glyphosate. Researchers discovered that the planned pilot-scale biobed could completely remove glyphosate, atrazine, carbendazim, and prometryn from wastewater with high pesticide concentrations after 180 days.

The target pollutant in the current investigation was chlorpyrifos (O,O-diethyl O-3,5,6-trichloro-2-pyridylphosphorothioate) due to its detrimental effects on several environmental phases. Kumari et al. (2008) observed that more than 80% of water samples were contaminated with chlorpyrifos and its residues were above regulatory limits. According to the conclusions of Dar et al. (2019), Chiu et al. (2021), and Saengsanga and Phakratok (2023), chlorpyrifos had numerous applications worldwide, including as an insecticide, acaricide, and termiticide in homes, public health, and agriculture against a variety of pests, causing chlorpyrifos to contaminate various habitats, including soil, sediments, water, and air. Thus, ongoing studies are being carried out all over the world to create and develop practical and efficient ways to remove chlorpyrifos and related substances from different environmental phases. Hence, the current study’s objectives aimed to provide an outline of an environmentally friendly technology to solve pesticide point source pollution problem with special reference to chlorpyrifos and dealing with management of agricultural wastes. Experiments were conducted to assess the effectiveness of a biomixture consisting of soil, vermicompost, and wheat for chlorpyrifos removal, as well as the role of nutrition mineral source (NPK) as a stimulant for the modules was studied.

## Materials and methods

### Preparation of biomixture

Soil samples were collected from Kom Hamada, EL-Behera governorate, Egypt. Samples were collected from a depth of 0–30 cm, air-dried, and passed through 2-mm sieve and stored in plastic bags for experimental study and analysis. The particle size percentage of the soil was determined to identify soil texture by the hydrometer method (FAO, 1974). Soil pH and electrical conductivity (EC) were measured in soil paste extracts according to Page et al. (1982). Total carbonate was determined by calcimeter method (Nelson & Winter, 1982). Soil organic carbon was determined by wet-oxidation according to Walkley-Black method (Page et al., 1982). The amounts of available nitrogen, phosphorous, and potassium were determined by standard methods according to Page et al. (1982). The soil has pH 7.5, EC 1.2 dS m^−1^, organic carbon (OC) 10.47%, total nitrogen 3.36%, CN _ratio_ 3.12, sand 40%, silt 23%, and clay 37%; the soil texture was clay loam, Ca^++^ (23 meq/l), Mg^++^ (37 meq/l), Na^+^ (28 meq/l), CO_3_^−^ (2 meq/l), HCO_3_^−^ (6 meq/l), CL^−^ (20 meq/l), SO_4_^−^ (32 meq/l), and total CaCO_3_ (2.8%).

The straw sample was obtained from Kom Hamada, EL-Behera governorate, Egypt. The straw was cut using a food processor to obtain small fragment approximately 3 mm to obtain homogeneous biomixture. The straw has 77% of organic carbon, and 2.8% total nitrogen (Page et al., 1982).

Some physic-chemical parameters of the commercial vermicompost were observed according to methodology described in Page et al. (1982). The vermicompost has EC 2.66 dS m^−1^, organic carbon 44.89%, and total nitrogen 1.75%.

The biomixture (B) was prepared according to Fernández-Alberti et al. (2012), Adak et al. (2020), and Fernandez et al. (2022) with some modifications. Vermicompost, wheat straw, and soil (without chlorpyrifos application history) were combined in a 1:2:1 volumetric ratio to create the laboratory scale biomixture. With using of distilled water, the mixture’s moisture content was adjust to 60% of its water holding capacity (WHC). Then the mixture was transferred to 1000-ml bottles and incubated in an incubator (Heraeus) in dark at 28 °C ± 2 for 45 days.

### Stimulation of biobeds

The influence of inorganic fertilizer (NPK) on the removal efficiencies of the tested biomixture (B) and soil (S) modules was studied according to Tortella et al. (2010). In order to obtain the stimulated biomixture (BF) or stimulated soil (SF), 200 g of the biomixture or soil samples (B or S) was put in glass jars and treated with 1% NPK fertilizer as a nutrient source.

### Experimental layout

Two hundred grams of each module (either B, BF, S, and SF) was separately added to glass jars. The moisture content was kept along for the experimental period at 60% of water-holding capacity by adding distilled water if needed. The four tested modules were spiked with 160 µg g^−1^ of chlorpyrifos in three cycles as follows:

I. The first cycle: technical grade chlorpyrifos (98%) was used and this cycle continues for 7 weeks.
II. The second cycle: formulated chlorpyrifos (48%) was added as fortification treatments to the same previous modules and this cycle continues for 8 weeks.
III. The third cycle: formulated chlorpyrifos (48%) was added as fortification treatments to the same previous modules and this cycle continues for 7 weeks.

**Fig. 1:**
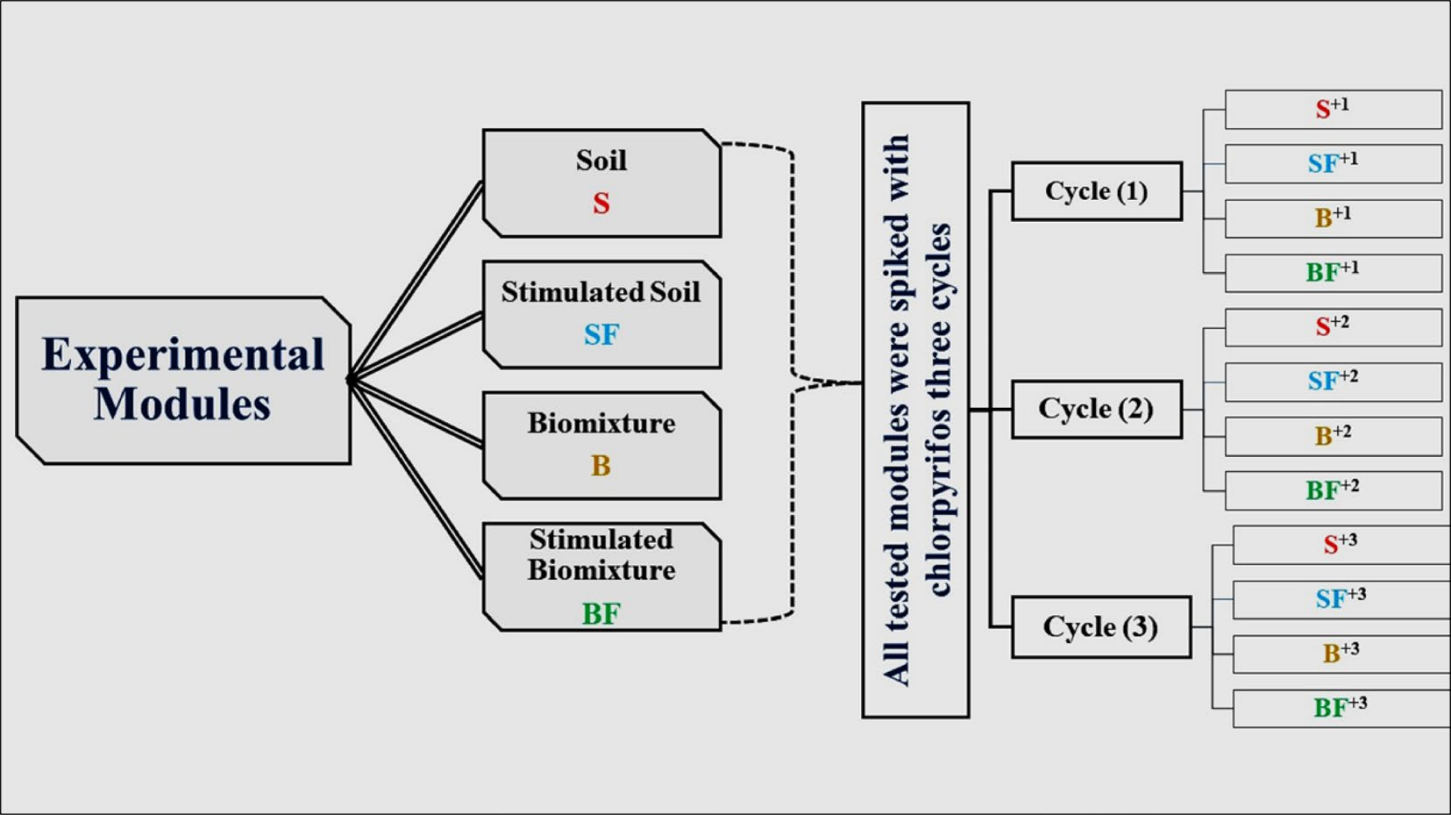
Treatments of tested modules

Each treatment was carried out in triplicate and incubated at 30 °C in laboratory incubator (Heraeus). On the 22nd -week of experimental period of all tested modules illustrated in Fig. 1, there was determination of the chlorpyrifos residue, dehydrogenase activity, and microbial respiration to study the potential of each module on removing chlorpyrifos as well as monitoring  the effect of chlorpyrifos on the microbial activity.

#### Extraction of chlorpyrifos from biomixture and soil samples

Chlorpyrifos extraction was carried out according to Wang et al. (2017) and Hala et al. (2021). Five grams from each module was taken at the interval times 0, 1, 2, 3, 4, 5, 6, and 7 weeks in the first treatment; 0, 3, 7, and 8 weeks in the second treatment; and 0 and 7 weeks in the third treatment. Then the sample was shaken (orbital shaker-JSSI-100 T-JSR) with 25 ml of acetonitrile:water (90:10, v/v) for 1 h at room temperature and centrifuged (Labofuge 200-Heraeus-SEPATECH) for 5 min at 4000 rpm. The supernatant was filtrated through activated charcoal powder layer on Whatman filter paper. Simultaneously, recovery (extract at time zero) and control experiments (module without chlorpyriphos) were conducted as a background correction. Concentration of the chlorpyrifos in the filtered supernatant was determined by measuring the absorbance using UV–visible light spectrophotometer (Alpha 1502-Laxco, Inc., Bothell, WA 98021, USA) at the optimum wavelength.

To obtain the optimum wavelength (λ max) for chlorpyrifos photometric determination using a UV–Vis spectrophotometer (Alpha 1502-Laxco, Inc., Bothell, WA 98021, USA), a spectral density curve (SD curve) was obtained by plotting chlorpyrifos optical density (O.D) versus wavelength (range 250–310 nm). Consequently, the 290-nm absorption was used in the present study for the quantitation of chlorpyrifos. The calibration standard curve (CD curve) was obtained by plotting different chlorpyrifos concentrations (5 to 80 µg mL^−1^) at the λmax (290 nm) wavelength. The calibration graph was linear (*R*^2^ = 0.972) up to a concentration range of 5–80 µg mL^−1^ which is sufficient for the current study.

#### Assay of dehydrogenase activity

Dehydrogenase activity was determined by 2,3,5-triphenyl tetrazolium chloride (TTC) as described by Casida et al. (1964) and Page et al. (1982). Briefly, 20 g of air-dried sample (< 2 mm) and 0.2 g of CaCO_3_ were mixed, and then, 6 g of this mixture was placed in test tubes, and 1 ml of a 3% aqueous solution of TTCand 2.5 ml of distilled water were added to the mixture. The tubes were stoppered and incubated at 37 °C. After 24 h, the stopper was removed and 10 ml methanol was added to each tube. After shaking for 1 min, the reaction mixture was filtrated through a glass funnel plugged with absorbent cotton into a 100-ml volumetric flask. All assays were done in 3 replicates. The intensity of the reddish color of the reaction mixture (final volume 100 ml) was measured by using a spectrophotometer (Alpha 1502-Laxco, Inc., Bothell, WA 98021, USA) at wavelength 485 nm.

#### Microbial respiration

The methodology outlined by Page et al. (1982) and Fernández-Alberti et al. (2012) was followed to conduct the microbial respiration assessment. Sample (100 g) from each module (60% WHC) was added to a one litter bottle, and small test tube containing 20 ml of NaOH (2N) was held in the bottle, and then tightly covered to avoid the reaction of NaOH with CO_2_. Samples were incubated at 28 °C for the aforementioned intervals. The respiration was expressed as milligrams of CO_2_/100 g sample. The estimated volume of released CO_2_ form organic compound decomposition was measured by titration NaOH (2 N) with HCl (0.2 N) after addition of BaCl_2_ and some drops of phenolphthalein as an indicator for determination end point of the reaction between HCl and NaOH by changing their color from pink to colorless.

### Consortia isolation from tested modules

This experiment was conducted according to Lu et al. (2013) and Mansee et al. (2020) with some modification. Five grams from each tested module (S, S^+3^, SF^+3^, B, B^+3^, and BF^+3^) was suspended in 50 ml Luria–Bertani )LB(medium (prepared by dissolving the following as g L^−1^ of distilled water: tryptone (10), yeast extract (5), and NaCl (10)), and then the mixture was incubated at 30 °C for 2 days in an orbital shaker (JSSI-100 T (JSR) at 180 rpm. One milliliter of each culture was subcultured into fresh L.B. media for 2 days under the same conditions. Finally, the cell pellets were collected by centrifugation at 5000 rpm for 5 min at room temperature and washed twice with sterilized mineral salt media (MSM) without any carbon source (prepared by dissolving the following as g L^−1^ of distilled water: NH_4_NO_3_ (1), K_2_HPO_4_ (1.5), KH_2_PO_4_ (0.5), NaCl (0.5), MgSO_4_ (0.2), and pH 7.0 (pH meter-3310 JENWAY)) and adjusted to approximately (2 × 10^8^ CFU mL^−1^) by previous media.

### Effect of chlorpyrifos on consortium growth

The potential of consortiums isolated from S, S^+3^, SF^+3^, B, B^+3^, and BF^+3^ modules (“Consortia isolation from tested modules” section) for using chlorpyrifos as a sole carbon source was studied according to Mansee et al. (2020) with few modifications. One hundred microliters of each isolated consortium was inoculated separately in 10 ml MSM supplemented with chlorpyrifos as a carbon source (prepared by dissolving the following as g L^−1^ of distilled water: NH_4_NO_3_ (1), K_2_HPO_4_ (1.5), KH_2_PO_4_ (0.5), NaCl (0.5), MgSO_4_ (0.2), 100 mg L^−1^ chlorpyrifos and adjust pH at 7.0). The control media was MSM without any carbon source (prepared by dissolving the following as g L^−1^ of distilled water: NH_4_NO_3_ (1), K_2_HPO_4_ (1.5), KH_2_PO_4_ (0.5), NaCl (0.5), MgSO_4_ (0.2), and pH 7.0) and MSM supplemented with glucose (prepared by dissolving the following as g L^−1^ of distilled water: NH_4_NO_3_ (1), K_2_HPO_4_ (1.5), KH_2_PO_4_ (0.5), NaCl (0.5), MgSO_4_ (0.2), 100 mg L^−1^ glucose and pH 7.0). Each treatment was carried out in triplicate. The culture samples were shaking in an orbital shaker at 180 rpm and 30 °C. The absorbance at 600 nm was used to calculate the turbidity at different interval times (0, 2, 4, 6, and 24 h) using UV spectrophotometer.

### Degradation of chlorpyrifos by consortiums in liquid culture

The efficiency of six isolated consorts (S_consortium_, S^+3^_consortium_, SF^+3^_consortium_, B_consortium_, B^+3^_consortium_, and BF^+3^_consortium_) for degrading chlorpyrifos was examined using the methodology of Lu et al. (2013). In 10 ml of MSM with 100 µg mL^−1^ of chlorpyrifos as the only carbon source, the degradation of the pesticide was conducted. Each treatment was performed in triplicate. After adding 100 µl of consortium cultures to MSM, the final cell concentration was achieved at about 2 × 10^6^ CFU ml^−1^. The mixture was then incubated at 30°C in a shaking incubator at 180 rpm. Cultures were routinely sampled every 2, 4, 6, and 24 h. Using a UV–visible spectrophotometer, the absorbance of the sample at 290 nm was measured to determine the remaining chlorpyrifos in each treatment.

### Statically analysis

Every data that were displayed stated as the mean ± standard deviation (SD) of the mean. Every piece of data represents an average across at least three separate studies. SAS software was used for all statistical analysis (SAS, Institutes Inc, 2007).

## Results and discussion

### Biomixture physicochemical properties

The current biomixture module was composed from soil, vermicompost, and wheat straw in the volumetric proportions of 1:1:2, respectively, as mentioned earlier, according to Góngora-Echeverría et al. (2018). The physicochemical characteristics of biomixture and soil were analyzed and are illustrated in Table 1. The variation between C/N_ratio_ values in biomixture (37) and soil (3.12) could be related to the homogeneity, the characteristics of the biomixture parent materials, and the incubation period. Castillo et al. (2008) concluded that there were a relation between the C/Nratio and pesticide removal. Thus, they recommended to replace the biomixtures when the C/N_ratio_ decreases to normal values of soil. Also, Castillo and Torstensson (2007) recommended a C/N_ratio_ of 30 for a straw-peat-soil (2:1:1) biomixture which is comparable to the current obtained ratio (37). On the other hand, Fernández-Alberti et al. (2012) investigated the relation between the biomixture humidity value and its biological activity and they observed that the biomixture with 60% of water holding capacity was preferred to express the highest degradation of chlorpyrifos. Also, Delgado-Moreno et al. (2017) studied the effects of different organic substances including vermicompost on the efficacy of biomixture for pesticides removing. They found that the biomixture containing vermicompost achieved the highest pesticide dissipation capacity. Hence, the suggested biomixture with characteristics pH 7.2, EC 1.8 dSm^−1^, WHC 60%, OC 49.38%, TN 1.33%, and C/N_ratio_ 37 is suitable for chlorpyrifos dissipation and in agreement with those introduced by several investigations (Castillo & Torstensson, 2007; Fernández-Alberti et al. 2012; Delgado-Moreno et al. 2017).

**Table 1:** Physicochemical properties of biomixture and soil modules

	Physicochemical properties				
Tested model	pH	EC (dSm^−1^)	O.C. (%)	T.N. (%)	C/N_ratio_
Soil	7.5	1.2	1.047	0.336	3.12
Biomixture	7.2	1.8	49.38	1.33	37

### The potential of biobed modules for chlorpyrifos dissipation

**Fig. 2:**
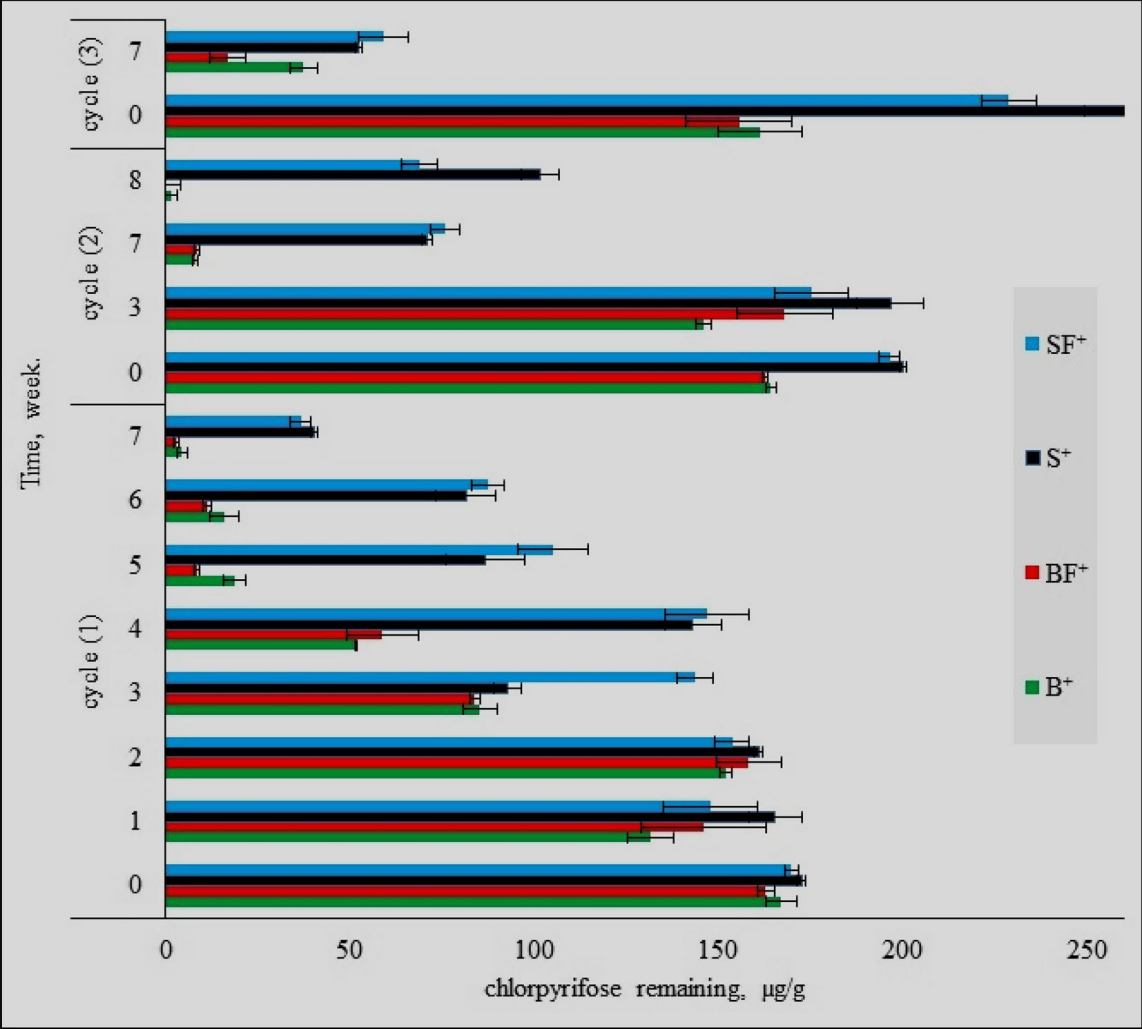
Chlorpyrifos remaining (µg g^−1^) in the biomixture and soil modules during 3 cycles of chlorpyrifos application

The purpose of these experiments was to evaluate the ability of biomixture and soil modules (B & S) to remove frequent additions of chlorpyrifos. Also, the effect of the NPK stimulator on the efficacy of biomixture and soil modules (BF& SF) was studied by fortifying both modules with inorganic fertilizer NPK at the rate of 1% (w/w) as described by Tortella et al. (2010). The dissipation rate of chlorpyrifos is determined in tested modules (S^+1^, SF^+1^, S^+2^, SF^+2^, S^+3^, SF^+3^, B^+1^, BF^+1^, B^+2^, BF^+2^, B^+3^, and BF^+3^) by measuring the remaining chlorpyrifos using a spectrophotometer at 290 nm at certain intervals (Fig. 2).

For the first application chlorpyrifos cycle, the results exemplify a significant difference between biomixture and soil strength in removing chlorpyrifos after 7 weeks. In biomixture modules, the chlorpyrifos residues decreased from 160 to 4.40 µg g^−1^ (approximately 97% dissipation). However, in the soil modules, the residual of chlorpyrifos decreased from 160 to 40.40 µg g^−1^ (approximately 75% dissipation). When studying the role of the NPK as an enhancing factor of stimulated modules at the end of the first cycle compared to the unstimulated modules, it was found that the stimulation process improved the pesticide removal rate from 97.25 to 98.13% (approximately 0.88% enhancement) at the end of the first cycle, whereas the soil modules increased the pesticide removal rate from 74.75 to 77.1% (approximately 2.35% enhancement) at the end of the same cycle. For the second cycle on the same modules, the results of tested biomixture and soil modules powerful for removing chlorpyrifos show a significant differences in chlorpyrifos dissipation when B^+2^ was used compared to S^+2^. The strength of the B^+2^ biomixture for removing chlorpyrifos after 8 weeks was observed when its residues decreased from 164.40 to 1.32 µg g^−1^ (approximately 99% dissipation). However, in S^+2^ modules, the chlorpyrifos residues decreased from 200.40 to 101.69 µg g^−1^ (approximately 49% dissipation). For the third cycle of chlorpyrifos addition, the dissipation rate due to using biomixture (B^+3^& BF^+3^) and soil (S^+3^& SF^+3^) modules was monitored. The obtained results showed the strength of B^+3^ for removing chlorpyrifos after 7 weeks when its residues decreased from 161.30 to 37.36 µg g^−1^. The role of the NPK as a stimulator for the BF^+3^ modules at the end of the third cycle when compared to the unstimulated modules showed an improvement in removing chlorpyrifos from 76.84 to 89.22% (approximately 12% enhancing). However, the same stimulator had a negative effect on removing chlorpyrifos between S^+3^ and SF^+3^ (79.99 to 74.08%). Finally, the obtained data observed that the BF^+3^ modules presented the best option for chlorpyrifos removal compared to other modules (B^+3^, S^+3^, and SF^+3^). Generally, the obtained data as illustrated in Fig. 2 showed that the highest chlorpyrifos removal capacity is usually recorded when the stimulated biomixture modules are used.

**Fig. 3:**
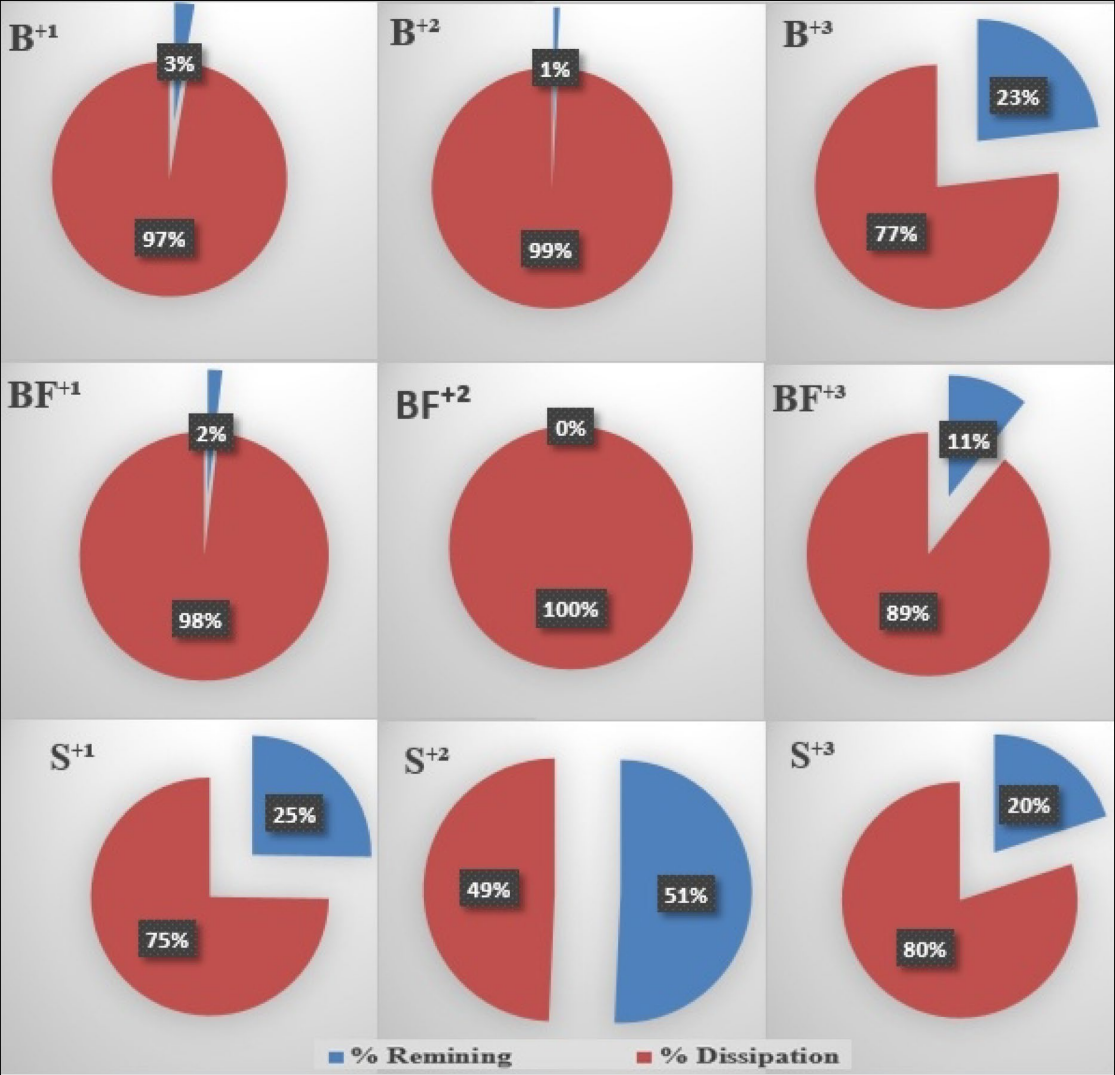
Chlorpyrifos dissipation at the end of three cycles of application

Figure 3 illustrates and summarizes the efficiency of the tested modules for removing frequent additions of chlorpyrifos from artificially contaminated environmental phases. Such results showed that the soil modules’ efficiency for removing chlorpyrifos was lower than that of biomixture modules. Also, chlorpyrifos almost completely disappeared from the biomixture samples after the first and second cycles of application. However, a slight reduction in the efficiency of biomixture modules was noticed after the third cycle of chlorpyrifos application. Results clarify that at the ends of the three cycles, the chlorpyrifos residues are higher in the soil modules than those in biomixture modules.

### Effects of chlorpyrifos on the biological activities

The changes in biomixture and soil quality during three cycles of chlorpyrifos addition were evaluated throughout the analysis of microbial respiration rate and dehydrogenase activities as bioindicators for the biological activities. These bioindicators were chosen according to Adesodun et al. (2005) to assess the status of microorganisms in the suggested modules.

#### Microbial respiration rate

**Fig. 4:**
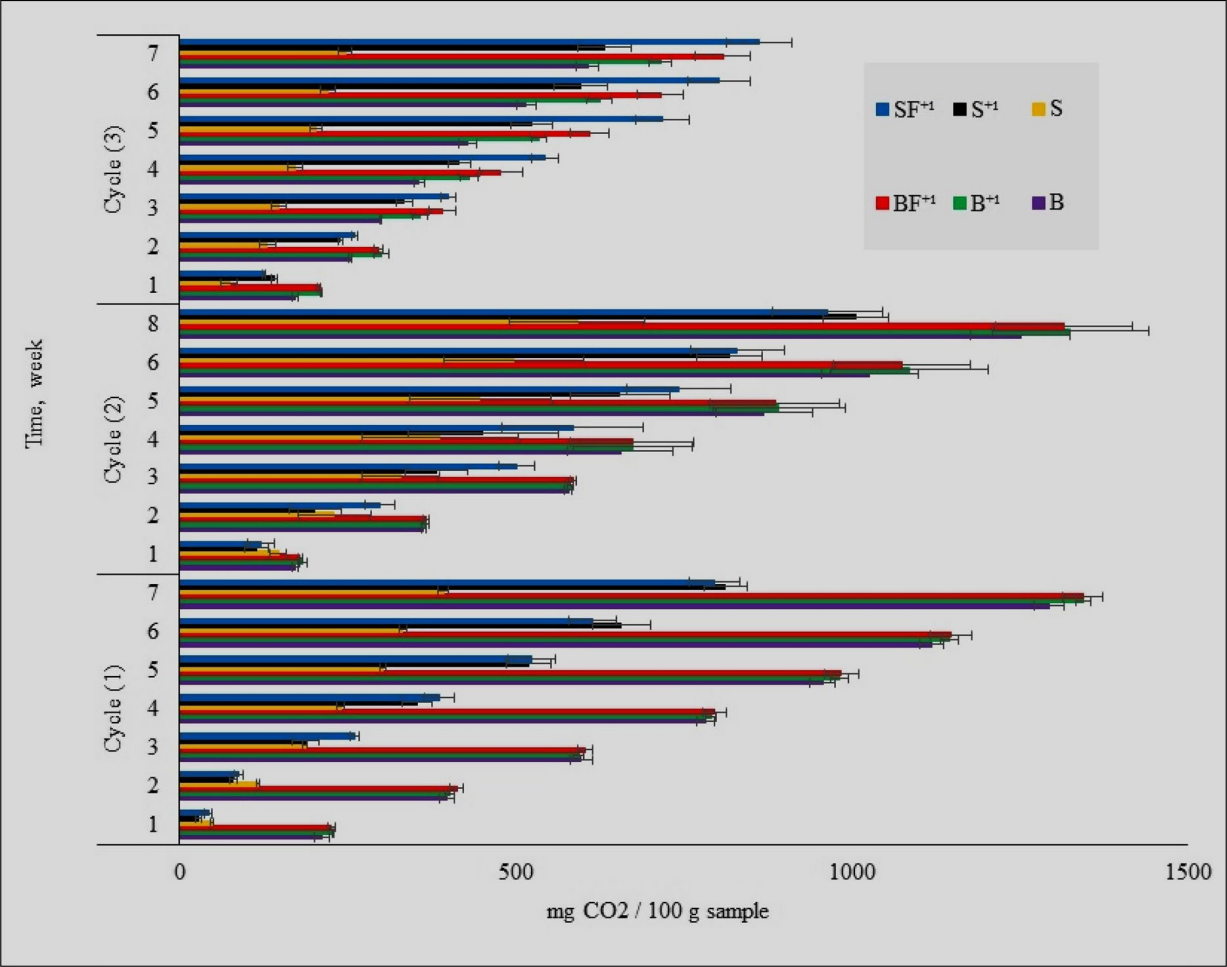
Effect of chlorpyrifos consequence three added cycles on accumulative CO_2_ emission from the current six studied modules

Figure 4 summarizes the effect of the repeated addition of the tested pesticide on the CO_2_ emission rate from the studied modules. The microbial respiration was determined by measuring CO_2_ evolution both in pesticide-treated modules (S^+^, SF^+^, B^+^, BF^+^) and corresponding untreated modules (S, B) at interval times in parallel with the chlorpyrifos dissipation during three cycles of chlorpyrifos application according to Fernández-Alberti et al. (2012). The accumulative CO_2_ emission from all tested modules clarified that there were a significant differences in the rate of carbon dioxide emission between biomixture and soil modules almost at all tested intervals times. Current results elucidate that CO_2_ emission increased in biomixture modules than those in the soil one. The role of integration between organic and mineral fertilizers represented in the stimulated modules partly enhanced CO_2_ emission in biomixture modules than those of the soil one. This is consistent with the results obtained in the section of pesticide dissipation by comparing the rate of chlorpyrifos dissipation in both soil and biomixture modules. Castillo and Torstensson (2007) found that pesticide dissipation and microbial respiration in peat-biomixtures are both positively correlated. Also, the effect of chlorpyrifos on soil respiration was studied by Dutta et al. (2010), and they found that the soil respiration rate decreased when the chlorpyrifos dose was increased from 0.5 to 50 mg kg^−1^ soil. Fernández-Alberti et al. (2012) studied the rate of CO_2_ production from contaminated and uncontaminated biomixture. They observed that no significant differences between the CO_2_ production from either contaminated and uncontaminated biomixture which related to the degradation of biomixture components provide a readily available nutrient sources, hence increment proliferation of the microorganisms. Also, Omirou et al. (2012) studied the dissipation of pesticides used during citrus production in either compost-based biomixtures, soil, or straw-soil mixture. They observed a significant correlation between microbial respiration and pesticide dissipation rates.

#### Dehydrogenase activity

**Fig. 5:**
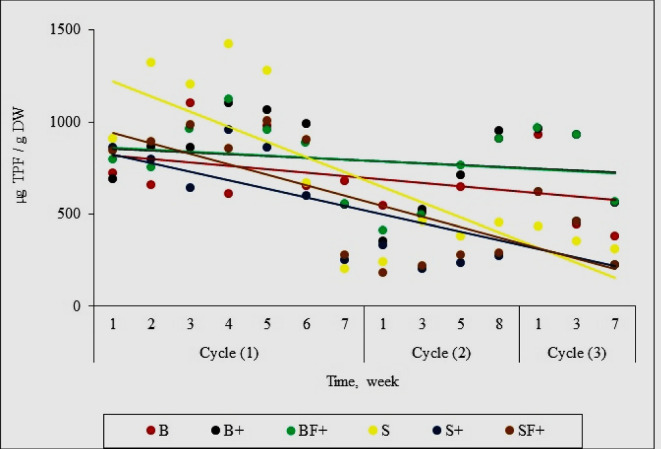
Effects of chlorpyrifos sequential additions on dehydrogenase activity for tested modules

Determination of dehydrogenase enzyme is one of the most adequate, important, and sensitive bio-indicators in soil systems and has been considered an indicator of overall microbial activity in soil (Romero et al. 2019). The dehydrogenase activity was determined in parallel with the chlorpyrifos dissipation to evaluate the impact consequence additions of chlorpyrifos on the biological system in the tested modules. Through the results shown in Fig. 5, it was noticed that the trend of dehydrogenase activity in soil modules (either stimulated or un stimulated) was decreased with time, while the trend of its activity in biomixture modules was almost constant despite repeated additions of the chlorpyrifos. In addition, the best module that tolerates repeated chlorpyrifos application is the stimulated biomixture module (BF^+^). This confirms the enhancement role of integration between biomixture and mineral fertilizer on the microbial activities and thus the ability to get rid of pollutants which emphasizes the study’s main objective. According to Sanchez-Hernandez et al. (2018), the decrease in chlorpyrifos concentration led to an increase in soil enzyme activity (dehydrogenase) that was higher than previous records before the pesticide application. Also, Wang et al. (2013) and Riah et al. (2014) reported that the use of dehydrogenase activity as a redox indicator of soil microorganisms is an important indicator of soil pollution levels.

### Potential of bacterial consortia for degrading chlorpyrifos

Isolation of bacterial consortium (S_consortium_, S^+3^_consortium_, SF^+3^_consortium_, B_consortium_, B^+3^_consortium_, and BF^+3^_consortium_) from the six studied modules (S, S^+3^, SF^+3^, B, B^+3^, and BF^+3^) after the third cycles of chlorpyrifos application was conducted to explore the relation between the studied treatments (stimulation and frequent addition of chlorpyrifos) and the biological efficiency. Also, such isolates were tested for its potential on removing chlorpyrifos by using it as the exclusive source of carbon.

#### Measuring the growth of bacterial consortia using the turbidity method

**Fig. 6:**
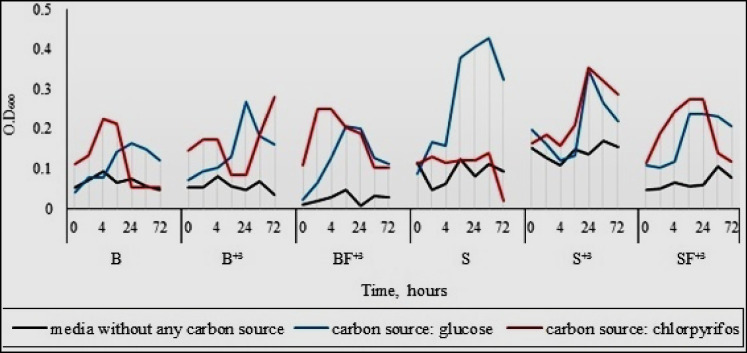
Growth of S_consortium_, S^+3^_consortium_, SF^+3^_consortium_, B_consortium_, B^+3^_consortium_, and BF^+3^_consortium_ consortia using chlorpyrifos or glucose as a sole carbon source

In this experiment, 2 × 10^6^ CFU/ml from each module consortium (S_consortium_, S^+3^_consortium_, SF^+3^_consortium_, B_consortium_, B^+3^_consortium_, and BF^+3^_consortium_) was inoculated in MSM containing 100 µg mL^−1^ of glucose or chlorpyrifos as a sole carbon source and incubated for 2, 4, 6, 24, 48, and 72 h, then the absorbance at 600 nm was measured as an indicator for consortium isolate growth. The result in Fig. 6 clarifies that the consortia S_consortium_ and S^+3^_consortium_ were almost inhibited in chlorpyrifos enrichment media. The studies by Kadian et al. (2012) and Góngora-Echeverría et al. (2018) found that organic amendments play a crucial role in maintaining the microbial community in chlorpyrifos-contaminated soil, and the chlorpyrifos-contaminated soil can be minimized with the application of organic amendments. This observation on the role of organic amendments for enhancing pollutant removal is consistent with our results that showed the efficiency of biomixture for chlorpyrifos removal.

#### The effectiveness of bacterial consortium for degrading chlorpyrifos

**Fig. 7:**
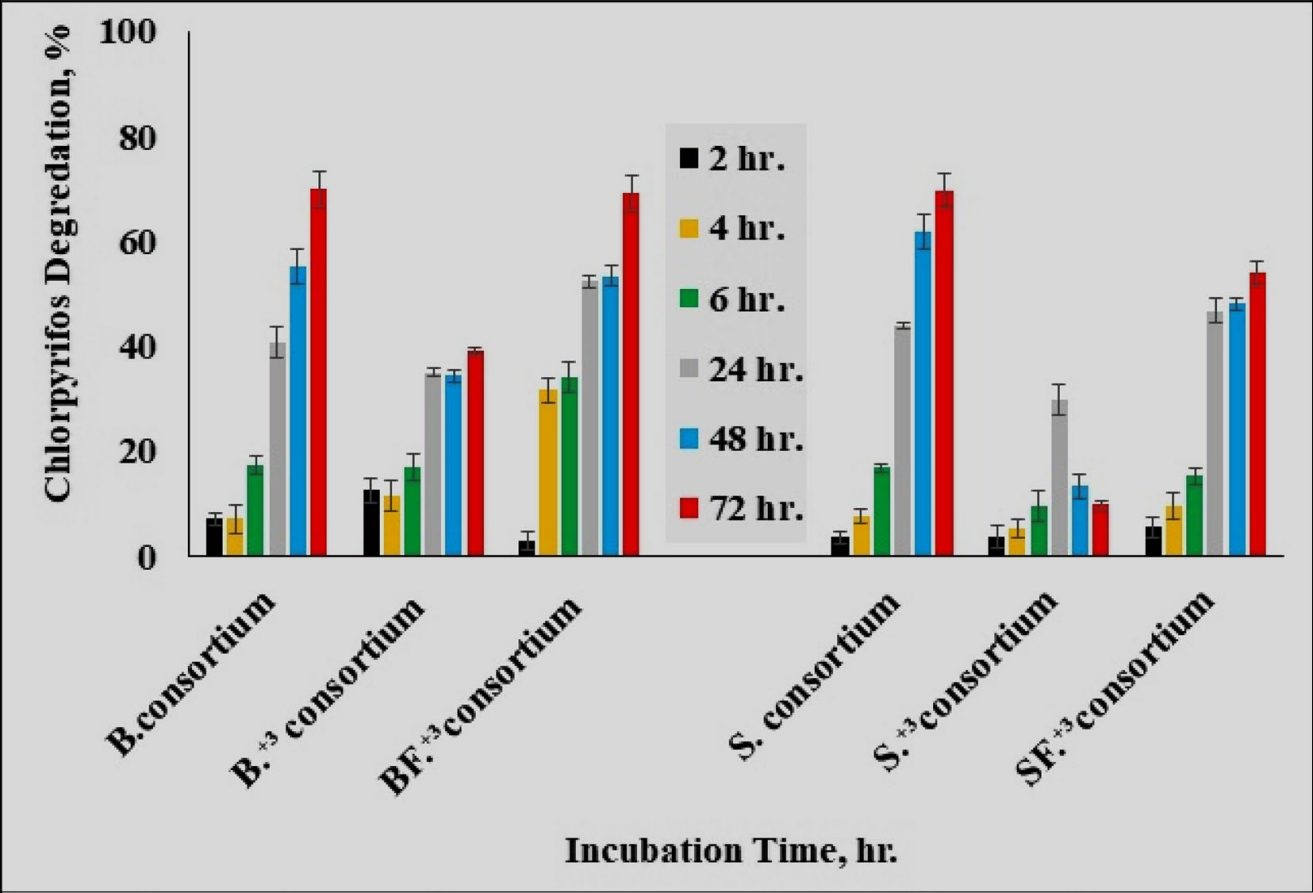
The behavior of current isolated consortiums in the presence of chlorpyrifos as a sole carbon source

The percentage of chlorpyrifos degradation was measured and calculated at the same previous intervals. Current results illustrated in Fig. 7 clarified that the consortiums isolated from B, BF^+3^, and S modules had more potential for degrading chlorpyrifos than other tested consortium isolates. The low efficiency of S^+3^_consortium_ (10%) for degradation chlorpyrifos compared to other consortiums may be due to the accumulation effect of chlorpyrifos multi-application to the tested soil, while biomixture construction could resist such accumulation effects. Góngora-Echeverría et al. (2018) reported that the physicochemical parameters of biomixtures affect the growth, diversity, and type of microorganisms which confirms the current finding. This may explain why S^+3^_consortium_ was less effective than the other tested consortiums. It could be observed that a reduction of consortia growth is sometimes combined with the higher chlorpyrifos degradation rate, this possibly caused by one or more chlorpyrifos metabolites emerged from degradation process and hence possibly affects the consortium isolates. Almost 24 h of incubation showed that the growth values were inhibited and the degradation rate increased in the case of B_consortium,_ B^+3^_consortium_, and S_consortium_. This finding may be due to some chlorpyrifos byproducts that negatively affect consortium growth rate. Also, from the present data, it can be noticed that there was no significant difference between the degradation percent with B_consortium_ and BF^+3^_consortium_ while the degradation efficiency of B^+3^_consortium_ was lower. This may be due to the positive impact of the NPK on BF^+3^ biological activities as compared to non-stimulated module B^+3^. According to Tortella et al. (2010), the presence of NPK in all evaluated concentrations (0.5 and 1.0%) led to an increase in biological activity in the biomixture, supporting these results.

## Conclusion

Selecting an excellent biomixture and the right circumstances is essential for managing pesticide contamination with biobeds technology. The biobed system’s performance is dependent on the biomixture’s proper preparation. The vermicompost and NPK help to eliminate chlorpyrifos’ inhibitory effect during the 22-week experimental period in current experimental modules. These findings were confirmed through current results as follows: the soil module (S^+^) was the lowest efficient in the chlorpyrifos degradation (49% removal) while the stimulated biomixture module (BF^+^) was the highest efficient module (100% removal). These findings were also confirmed when the efficiency of the isolated consortium to degrade chlorpyrifos was tested. It can be noticed that the best removal efficiency was achieved when testing the BF^+3^_consortium_ which was isolated from BF^+^ modules after three rounds of contamination with a chlorpyrifos addition to the B_consortium_ and S_consortium_ (isolated from untreated modules). Finally, it can be concluded that integration between vermicompost and NPK is expected to effectively display a high microbial activity as well as support the chlorpyrifos dissipation as compared to other experimental modules.

## Data Availability

The authors confirm that the data supporting the findings of this study are introduced and available within the manuscript.
